# 

**Published:** 1885-11

**Authors:** 


### EXCHANGES RECEIVED.

*American.*—Atlanta Medical and Surgical Journal. American Journal of Pharmacy. American Journal of Physiology. American Journal of Obstetrics. American Medical Digest. American Practitioner. American Journal of Ophthalmology. American Journal of Insanity. New York Medical Journal. American Journal of the Medical Sciences. American Journal of Dental Science. Archives of Pediatrics. Annals of Surgery. Analectic Medical Monthly. The Alienist and Neurologist. Buffalo Medical and Surgical Journal. Boston Medical and Surgical Journal. Cincinnati Lancet and Clinic. Columbus Medical Journal. College and Clinical Record. The Cincinnati Times. Dental Register. Detroit Lancet. Denver Medical Times. Ephemeris. Fort Wayne Journal of the Medical Sciences. Gailliard's Medical Monthly. Indiana Medical Journal. Index Medicus. Journal of the American Medical Association. Journal of Nervous and Mental Disease. Journal of the Materia Medica. Journal of Inebriety. Louisville Medical News. Medical Annals. Medical News. Maryland Medical Journal. Medical Record. Mississippi Valley Medical Monthly. Medical and Surgical Reporter. Medical Chronicle. Medical World. Medical Bulletin. Medical Age. Nashville Journal of Medicine and Surgery. New Orleans Medical and Surgical Journal. Northwestern Lancet. Obstetric Gazette. Philadelphia Medical Times. Peoria Medical Monthly. Pacific Medical and Surgical Journal. The Pharmacist. Southern Medical Record. Sanitarian. Southern Practitioner. St. Louis Medical and Surgical Journal. San Francisco Western Lancet. Southern Clinic. St. Louis Courier of Medicine. Sanitary News. Therapeutic Gazette. Virginia Medical Monthly. Weekly Medical Review. Western Medical Reporter. The Esculapian. Atlantic Journal of Medicine. Eastern Medical Jonrnal. The Physician's Monitor. The Polyclinic. The Texas Courier-Record of Medicine. The Kansas City Medical Record. The Puget Sound Sanitarian and Prohibitionist. New England Medical Monthly. California Medical Journal. The Iowa State Medical Reporter. The Kansas City Medical Index. Journal of Cutaneous and Venereal Diseases. St. Louis Medical Journal. The Western Medical Reporter. Quarterly Compendium of Medical Science. American Druggtst. The N. C. Medical Journal. Eastern Medical Journal.

*Canadian.*—Canada Medical and Surgical Journal. Canadian Practitioner. Canada Lancet. Therapeutic Gazette. L'Union Medicale du Canada.

*English.*—The Practitioner. Students' Journal and Hospital Gazette. British Medical Journal. Brain. Medical Press and Circular. Medical Chronicle. The London Lancet.

*Scotch.*—Edinburgh Medical Journal.

*French.*—Revue Medicale de l'est. Revue Medicale. Le Praticien. Le Progrés Medical. Bulletin de l'Academie de Medecine. Journal de L'Anatomie et de La Physiologie. Archives Generales de Medecine. Recueil D'Ophtalmologie. Lyon Medical La France Medicale. Journal de Medecine et de Chirurgie. Union Medicale et Scientifique du Nord-Est. La Medicine Contemporaine.

*German*—Wiener Medizinisch. Wochenschrift. Medicinisch-Chirurgisches Centralblatt. Der Irrenfreund. Memorabilien.

*Swiss*—Correspondenz. Blatt fur Schweizer Aerzte.

*Russian*—St. Petersburger Medizinische Wochenschrift.

*Italian.*—Revista Internazionale die Medicina e Cherurgia. La Sperimentales Giornale Internazionale delle Scienze Mediche.

*Spanish*—Revista de Ciencias Medicas La Independencia Medica.

*South American.*—(Buenos Ayres).—Revista Medico-Quirurgica. El Eüsayo Mèdico, Caracas.

*Australia.*—The Australian Medical Gazette, Sydney, Australia.

SUBSCRIPTION PRICE, $3.00 PER YEAR.

Whole Number, 311. NOVEMBER, 1885. Vol. LI., Number 5.

INDEX MEDICUS

LIBRARY INDEXED S. G. O.

### THE CHICAGO MEDICAL JOURNAL AND EXAMINER

EDITORS:

S. J. JONES, M.D., LL.D.

W. W. JAGGARD, A.M., M.D. HAROLD N. MOYER, M.D.

---

COLLABORATORS.

CHICAGO:

Professor J. A. ALLEN, M. D., LL. D.
Professor E. ANDREWS, M. D., LL. D.
Professor W. H. BYFORD, A. M., M. D.
Professor O. C. DeWOLF, A. M., M. D.
Professor E. C. DUDLEY, A. B., M. D.
Professor J. H. ETHERIDGE, A. M., M. D.
Professor C. FENGER, M. D.
Professor J. N. HYDE, A. M., M. D.
Professor E. F. INGALS, A. M., M.D.
Professor H. A. JOHNSON, M. D., LL. D.
CHARLES GILMAN SMITH, A. M., M.D
J. F. TODD, M. D.

MILWAUKEE, WISCONSIN:
Professor N. SENN, M. D.

WASHINGTON, D. C:
S. O. RICHEY, M. D.

UNITED STATES NAVY
MEDICAL DIRECTOR J. M. BROWNE, M. D.

CHICAGO:

